# A multicentre, efficacy and safety study of methotrexate to increase response rates in patients with uncontrolled gout receiving pegloticase (MIRROR): 12-month efficacy, safety, immunogenicity, and pharmacokinetic findings during long-term extension of an open-label study

**DOI:** 10.1186/s13075-022-02865-z

**Published:** 2022-08-25

**Authors:** John K. Botson, John R. P. Tesser, Ralph Bennett, Howard M. Kenney, Paul M. Peloso, Katie Obermeyer, Yang Song, Brian LaMoreaux, Lin Zhao, Yan Xin, Jason Chamberlain, Srini Ramanathan, Michael E. Weinblatt, Jeff Peterson

**Affiliations:** 1 Orthopedic Physicians Alaska, 3801 Lake Otis Parkway, Suite 300, Anchorage, AK 99508 USA; 2 Arizona Arthritis & Rheumatology Associates, 4550 East Bell Road, Phoenix, AZ 85032 USA; 3 Arthritis Northwest, PLLC, 105 West 8th Avenue, Suite 6080W, Spokane, WA 99204 USA; 4 Horizon Therapeutics Plc, 1 Horizon Way, Deerfield, IL 60015 USA; 5 Horizon Therapeutics Plc, 2 Tower Place, South San Francisco, CA 94080 USA; 6 Division of Rheumatology, Inflammation and Immunity, Brigham and Women’s Hospital, 75 Francis Street, Boston, MA 02115 USA; 7 Western Washington Medical Group Arthritis Clinic, 1909 214th Street SE, Suite 211, Bothell, WA 98021 USA

**Keywords:** Pegloticase, Methotrexate, Gout, Tophi

## Abstract

**Background:**

Publications suggest immunomodulation co-therapy improves responder rates in uncontrolled/refractory gout patients undergoing pegloticase treatment. The MIRROR open-label trial showed a 6-month pegloticase + methotrexate co-therapy responder rate of 79%, compared to an established 42% pegloticase monotherapy responder rate. Longer-term efficacy/safety data are presented here.

**Methods:**

Uncontrolled gout patients (serum urate [SU] ≥ 6 mg/dL and SU ≥ 6 mg/dL despite urate-lowering therapy [ULT], ULT intolerance, or functionally-limiting tophi) were included. Patients with immunocompromised status, G6PD deficiency, severe kidney disease, or methotrexate contraindication were excluded. Oral methotrexate (15 mg/week) and folic acid (1 mg/day) were administered 4 weeks before and during pegloticase therapy. Twelve-month responder rate (SU < 6 mg/dL for ≥ 80% during month 12), 52-week change from baseline in SU, and extended safety were examined. Efficacy analyses were performed for patients receiving ≥ 1 pegloticase infusion. Pharmacokinetics (PK)/anti-drug antibodies (ADAs) were examined and related to efficacy/safety findings.

**Results:**

Fourteen patients were included (all male, 49.3 ± 8.7 years, 13.8 ± 7.4-year gout history, pre-therapy SU 9.2 ± 2.5 mg/dL). Three patients were non-responders and discontinued study treatment before 24 weeks, one patient exited the study per protocol at 24 weeks (enrolled prior to treatment extension amendment), and 10 remained in the study through week 52. Of the 10, 8 completed 52 weeks of pegloticase + methotrexate and were 12-month responders. The remaining two discontinued pegloticase + methotrexate at week 24 (met treatment goals) and stayed in the study under observation (allopurinol prescribed at physicians’ discretion); one remained a responder at 12 months. At 52 weeks, change from baseline in SU was − 8.2 ± 4.1 mg/dL (SU 1.1 ± 2.4 mg/dL, *n* = 10). Gout flares were common early in treatment but progressively decreased while on therapy (weeks 1–12, 13/14 [92.9%]; weeks 36–52, 2/8 [25.0%]). One patient recovered from sepsis (serious AE). Two non-responders developed high ADA titers; fewer patients had trough concentrations (*C*_min_) below the quantitation limit (BQL), and the median *C*_min_ was higher (1.03 µg/mL vs. BQL) than pegloticase monotherapy trials.

**Conclusions:**

Pegloticase + methotrexate co-therapy was well-tolerated over 12 months, with sustained SU lowering, progressive gout flare reduction, and no new safety concerns. Antibody/PK findings suggest methotrexate attenuates ADA formation, coincident with higher treatment response rates.

**Trial registration:**

ClinicalTrials.gov, NCT03635957. Registered on 17 August 2018.

**Supplementary Information:**

The online version contains supplementary material available at 10.1186/s13075-022-02865-z.

## Background

Gout is a common, inflammatory arthritis caused by monosodium urate deposition in the setting of chronically elevated serum urate levels (SU > 6 mg/dL). Monosodium urate crystals cause chronic systemic inflammation, even between acute gout flares [1, 2]. Additionally, hyperuricemia has been linked to multiple comorbidities, including hypertension [3, 4], cardiovascular disease [5–9], diabetes [3, 10], kidney disease [11, 12], metabolic syndrome [13], and a higher mortality rate [9, 14, 15]. Gout treatment guidelines recommend maintaining SU below at least 6 mg/dL [16, 17], but some patients are unable to meet this target because of urate-lowering therapy (ULT) under-utilization [18], patient non-compliance [19, 20], ULT intolerance, or ULT inefficacy [21]. As a result, an estimated 10% of patients may develop uncontrolled gout [22], which markedly impairs patient quality of life and physical functioning [23].

Pegloticase, a pegylated uricase enzyme, is an FDA-approved therapy for adult patients with uncontrolled gout and is highly effective in lowering SU [24]. It should be noted that pegloticase is commercially available only in the USA. The recombinant enzyme rapidly lowers SU by converting uric acid to allantoin, a water-soluble molecule that is readily excreted by the kidneys. Though the initial response to treatment is robust, many patients develop anti-drug antibodies (ADAs) to the therapy and are unable to complete a full course of treatment. Clinical trials of pegloticase monotherapy found a 42% treatment responder rate (responder defined as a patient with SU < 6.0 mg/dL for 80% of the time or longer during both months 3 and 6 of therapy), with approximately one-quarter (26%) of patients experiencing infusion reactions (IRs) when a pre-infusion monitoring protocol for SU was not followed [24, 25]. Anti-pegloticase antibodies that develop following pegloticase exposure are associated with both loss of urate-lowering effect, through increased pegloticase clearance, and a higher risk of IRs [26–28]. Starting in 2017, successes with immunomodulation co-therapy have been reported in the real-world setting with methotrexate [29, 30] and leflunomide [31] and in a clinical trial setting with azathioprine (TRIPLE open-label) [32], methotrexate (MIRROR open-label trial) [33], and mycophenolate mofetil in a randomized placebo-controlled clinical trial (RECIPE trial) [34]. Likely related to these reports, immunomodulation use with pegloticase is increasing in the USA [35]. In 2015, approximately 2% of patients treated with pegloticase were also treated with immunomodulation, whereas in 2019, 15% of new pegloticase patients were also treated with immunomodulating co-therapy [35]. The initial MIRROR open-label clinical trial reports describe 6-month outcomes [33], but longer-term safety and efficacy have not yet been published. Here, 12-month efficacy outcomes are described, along with pharmacokinetic (PK) and immunogenicity findings.

## Methods

### Study population

This study population has been fully described elsewhere [33]. Briefly, patients with uncontrolled gout aged 18 − 65 years were considered for trial enrollment. Patients were said to have uncontrolled gout if their SU level was ≥ 6 mg/dL at screening and at least one of the following was true: they were unable to maintain SU < 6 mg/dL on an oral ULT, they had an intolerance to their current ULT, or tophaceous deposits that limited patient functionality were present (detected clinically or with dual-energy computed tomography [DECT]). The key exclusion criteria included serious acute bacterial infection < 2 weeks prior to screening, severe chronic/recurrent bacterial infection, immunocompromised status, glucose-6-phosphate dehydrogenase (G6PD) deficiency (tested at screening), severe chronic renal impairment (glomerular filtration rate [GFR] < 25 mL/min/1.73 m^2^ or currently on dialysis), or liver disease (alanine aminotransferase [ALT] or aspartate aminotransferase [AST] > 3 times upper limit).

### Study medications

All enrolled patients were scheduled to receive 4 weeks of oral methotrexate (15 mg/week, run-in period) followed by treatment with both pegloticase (8 mg infusion every 2 weeks) and methotrexate (15 mg orally every week) for up to 52 weeks (treatment period). As previously described in this study’s first publication reporting 6-month findings [33], the methotrexate dose was chosen based on the enhancement of other biologics’ durability when methotrexate was used as co-therapy, published rheumatology expert opinions, and study advisory board recommendations. The original protocol included a 24-week treatment period, but a protocol amendment extended the treatment period to 52 weeks. Patients also received 1 mg/day of oral folic acid during both the run-in and treatment periods. All patients were required to begin gout flare prophylaxis (colchicine, non-steroidal anti-inflammatory drugs [NSAIDs], and/or low-dose prednisone [≤ 10 mg/day] as chosen by the treating physician) at least 1 week prior to initiating pegloticase, continuing flare prophylaxis per American College of Rheumatology guidelines [16]. When they did occur, flares were managed with NSAIDs, colchicine, corticosteroids, and intraarticular steroid injections at the treating physician’s discretion.

Patients were administered standard IR prophylaxis prior to each pegloticase infusion. This included oral fexofenadine (60 or 180 mg) the day before and morning of infusion, acetaminophen (1000 mg) the morning of infusion, and intravenous glucocorticoid (200 mg hydrocortisone or 125 mg methylprednisolone) immediately prior to each infusion. An SU monitoring protocol [25] was implemented to decrease the risk of IRs. Patients discontinued pegloticase + methotrexate co-therapy if they had two consecutive SU measurements above 6 mg/dL after week 2.

### Study procedures

Study procedures from screening to month 6 have been previously described [33]. Briefly, screening included study eligibility confirmation and patient medical and surgical history (including concomitant medications), physical examination, gout assessment (including flare history), and laboratory testing (SU measurement, hematology, clinical chemistry). After eligibility was confirmed, patients began the 4-week methotrexate run-in period (− 4 weeks) within 2 weeks of screening and returned for physical, laboratory, gout flare, and safety assessment at week − 2.

Pegloticase therapy was initiated on day 1 (methotrexate and folic acid continued during pegloticase treatment) and had a maximum duration of 52 weeks. Patients returned every 2 weeks for follow-up assessment (concomitant medication update, physical examination) and pegloticase infusion [see Additional file 1]. Safety assessments at each study visit included adverse event (AE) evaluation and blood/urine sampling for laboratory measurement and SU monitoring. The published Rheumatology Common Terminology Criteria for Adverse Events (CTCAE) grading system (1 = mild, 2 = moderate, 3 = severe, 4 = life-threatening) was used to determine AE severity [36]. When gout flares were documented, severity was assessed using a standardized flare grading system based on joint pain, joint swelling, and pain levels at rest [37].

### Pharmacokinetics and anti-drug antibodies

Blood samples for pegloticase PK evaluations were collected before and after pegloticase infusion on day 1 and on weeks 4, 8, 22, and 36. Samples were also collected before pegloticase infusion at weeks 10, 14, 18, 22, and 52. Pre-infusion blood samples were collected from all patients to evaluate the immunogenicity of pegloticase by measuring both anti-PEG and anti-uricase immunoglobulin G (IgG) antibodies (ADAs) at day 1 and weeks 2, 4, 6, 8, 10, 14, 18, 22, 24, 36, and 52. A small subset of patients had additional visits at weeks 1 and 7 (non-infusion visits), during which blood samples were collected for both PK and antibody evaluations. Pre-infusion blood samples were also collected to measure methotrexate polyglutamate(s) (MTX-PGs 1 to 5) in red blood cells at day 1 and weeks 4, 8, 22, 24, and 36.

### Bioanalytical assays

Pegloticase concentration in the serum was measured using a validated enzymatic/fluorescence assay (Charles River Laboratories, Senneville, Quebec, Canada). The calibration range was 0.6 to 10 µg/mL. ADAs were measured using a validated enzyme-linked immunosorbent assay (anti-pegloticase/PEG, anti-uricase; Precision for Medicine, Redwood City, CA, USA). The drug tolerance level was < 0.02 µg/mL in neat serum. Measurements of MTX-PG1 to 5 red blood cell concentrations were performed by Exagen Inc. (AVISE® MTX test; Vista, CA, USA) using a liquid chromatography–mass spectrometry method [38].

### Pegloticase pharmacokinetic analysis

Serum pegloticase concentration data were analyzed using two methods to evaluate the impact of methotrexate co-therapy on pegloticase PK. First, pegloticase exposures (median observed peak concentration [*C*_max_] and trough concentration [*C*_min_] across visits) with methotrexate from the current study were compared to the observed values in historical monotherapy phase 3 studies (C0405 and C0406) [23, 26]. Second, the observed pegloticase concentrations with methotrexate co-therapy in the current study were overlaid against the 90% prediction band of pegloticase concentrations following monotherapy based on the population PK model from the phase 3 data [38]. Pharmacokinetic profiles of pegloticase following 8 mg IV infusion every 2 weeks for a total of 12 infusions were simulated for 400 patients with an average body surface area of 2.12 m^2^ (percent coefficient of variation = 13.3%). The population PK simulations were done using NONMEM 7.4 (ICON Development Solutions, Ellicott City, MD, USA).

### Statistical methods

The primary efficacy endpoint was the proportion of responders during month 6 (SU < 6 mg/dL for ≥ 80% of month 6) and has been fully reported elsewhere [33]. Secondary and exploratory efficacy objectives described here include the proportion of treatment responders (SU < 6 mg/dL during ≥ 80% of the examined time) during months 3, 9, and 12; overall response rate during months 3 and 6 combined; and mean change from baseline in SU at weeks 14, 24, 36, and 52. A sample size of 12–16 patients was planned, which would demonstrate a statistically greater response rate over pegloticase monotherapy if at least 10/13 (77%) patients were responders for the primary endpoint (proportion of pegloticase responders during month 6 in pegloticase pivotal trials [43.5%] [24]), based on an exact test for proportions with a 5% type 1 error. Longer-term (9 months, 12 months) efficacy endpoints do not have established historical comparators.

All efficacy and safety analyses were performed on the modified intent-to-treat (mITT) population, defined as all patients who received at least 1 dose of pegloticase. Continuous variables are summarized by visit as mean ± standard deviation, and categorical variables are summarized as *n* (%). SU values below quantification limits (BQL) were set to zero for analyses. Safety analyses were also performed on data collected during the run-in period using the ITT population, defined as all patients who received at least 1 dose of methotrexate. The incidence and titer of positive ADAs (anti-PEG and anti-uricase) are summarized by visit.

## Results

Seventeen patients were screened for study eligibility between September 2018 and April 2019. Fifteen patients began the methotrexate run-in period and made up the ITT population. Fourteen completed the entire 4-week run-in period (1 patient was lost to follow-up after week − 2), began pegloticase + methotrexate co-therapy, and made up the mITT population through week 24. As already reported, 11 of 14 completed 24 weeks of pegloticase + methotrexate co-therapy for a 6-month responder rate of 79% (95% CI 49.2 to 95.3%; 3 patients discontinued pegloticase prior to 24 weeks because of SU rise) [33]. Of the 11 patients remaining in the study at week 24, 8 continued therapy through week 52, 2 met gout treatment goals (as determined by the treating investigator) at week 24 discontinuing study treatment but remaining in the study for observation through week 52, and 1 completed the study at week 24 (enrolled prior to protocol amendment that extended treatment). One of the patients who met the treatment goals at week 24 initiated allopurinol at the treating investigator’s discretion at week 24 (300 mg/day for 1 week, 600 mg/day for 2 weeks, then 300 mg/day through week 52), and the other patient started allopurinol at week 26 (150 mg/day for 21 weeks then 300 mg/day through week 52 [see Additional file 2]). The patient who completed the study at week 24 was not included in the analyses beyond 24 weeks (i.e., mITT *N* = 13 after week 24).

Table 1 summarizes the mITT patient characteristics at baseline. Briefly, patients were 49.3 ± 8.7 years of age and had an average gout history of 13.8 ± 7.4 years. Prior to initiating pegloticase, SU averaged 9.2 ± 2.5 mg/dL and 13 patients (93%) had clinically evident tophi.

**Table 1:** Baseline characteristics for the modified intent-to-treat population (*N* = 14)

Age, mean ± SD, years	49.3 ± 8.7
Male sex, *n* (%)	14 (100)
Race, *n* (%)	
White	12 (85.7)
Asian	2 (14.3)
Body mass index (BMI), mean ± SD, kg/m^2^	33.9 ± 7.0
Smoking status, *n* (%)	
Never	4 (28.6)
Current	5 (35.7)
Former	5 (35.7)
Gout characteristics	
Time since first gout diagnosis, mean ± SD, years	13.8 ± 7.4
Number of gout flares in the 12 months prior to Screening, mean ± SD	10.8 ± 8.5
History of tophi, *n* (%)	13 (92.9)
Baseline serum urate, mean ± SD, mg/dL	9.2 ± 2.5

### Efficacy outcomes

**Fig. 1:**
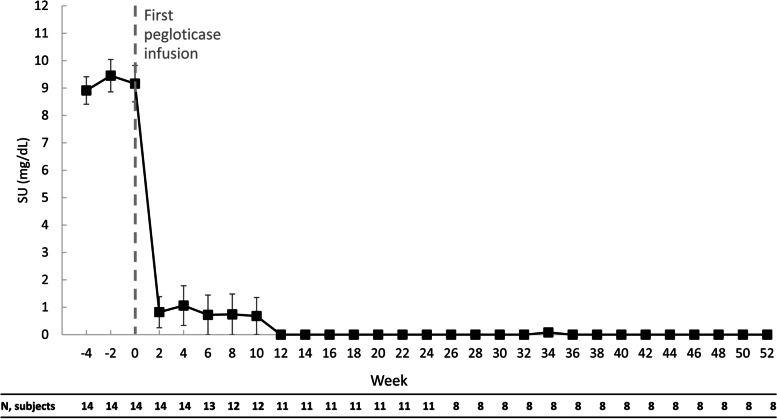
Pre-infusion serum urate levels during the methotrexate run-in (4 weeks) and pegloticase + methotrexate treatment (up to 52 weeks) periods. In the 3 non-responders, serum urate (SU) increases above 6 mg/dL were noted at weeks 2 and 4, weeks 4 and 6, and weeks 8 and 10. Data points represent the mean values, and error bars represent standard error (includes patients on treatment, values below the lower limit of detection were set to 0). SU, serum urate

All 8 patients who continued pegloticase + methotrexate co-therapy through week 52 were treatment responders during months 9 and 12. Pegloticase response rate in the mITT population was 10 of 13 patients (76.9%) during month 9 and 9 of 13 patients (69.2%) during month 12 (Table 2). Of the 4 patients who did not meet the response criteria during month 12, all had discontinued study treatment prior to (3 patients met the pegloticase discontinuation criteria [1 patient each at week 4, week 6, and week 10]) or at week 24 (1 patient met the treatment goals and discontinued pegloticase). After initiating pegloticase, SU rapidly decreased and was 0.0 ± 0.0 mg/dL at week 24 (change from baseline − 9.3 ± 2.8 mg/dL, *n* = 11) and 1.1 ± 2.5 mg/dL at week 52 (change from baseline − 8.2 ± 4.1 mg/dL, *n* = 10). In the patients remaining on pegloticase + methotrexate treatment through week 52 (*n* = 8), SU was 0.0 ± 0.0 at weeks 36 and 52 (change from baseline − 9.4 ± 3.3 mg/dL; Fig. 1, Table 2). In the 2 patients who remained in the study, but discontinued pegloticase + methotrexate at week 24, SU remained < 6 mg/dL until week 36 in the patient who initiated allopurinol at week 24 and until week 50 in the patient who initiated allopurinol at week 26 [see Additional file 2].

**Table 2:** Efficacy endpoints through month 12/week 52

Efficacy endpoint	mITT population
Maintained SU < 6 mg/dL for at least 80% of the time during	
Month 3, *n* (%) [95% CI], *N* = 14	11 (78.6%) [49.2 − 95.3]
Month 6, *n* (%) [95% CI], *N* = 14	11 (78.6%) [49.2 − 95.3]
Months 3 and 6 (overall), *n* (%) [95% CI], *N* = 14	11 (78.6%) [49.2 − 95.3]
Month 9, *n* (%) [95% CI], *N* = 13	10 (76.9%) [46.2 − 95.0]
Month 12, *n* (%) [95% CI], *N* = 13	9 (69.2%) [38.6 − 90.9]
SU change from baseline, mg/dL, mean ± SD (median [min, max])^a^	
Week 24, mean ± SD (median [min, max]), *n* = 11	− 9.3 ± 2.8 (− 9.1 [− 15.8, − 4.7])
Week 36, mean ± SD (median [min, max]), *n* = 8^b^	− 9.4 ± 3.3 (− 9.3 [− 15.8, − 4.7])
Week 52, mean ± SD (median [min, max]) *n* = 8^b^	− 9.4 ± 3.3 (− 9.3 [− 15.8, − 4.7])

Confidence intervals (CI) based on exact Clopper-Pearson CI

*mITT* Modified intent to treat (all patients exposed to pegloticase), *SU* Serum urate

^a^ Includes patients remaining on treatment; values below the lower limit of detection were set to 0; baseline is the last observation prior to the first pegloticase infusion

^b^ Change from baseline was − 8.1 ± 4.0 mg/dL at week 36 and − 8.2 ± 4.1 mg/dL at week 52 for all 10 patients remaining in the study through week 52

### Pharmacokinetics

**Fig. 2:**
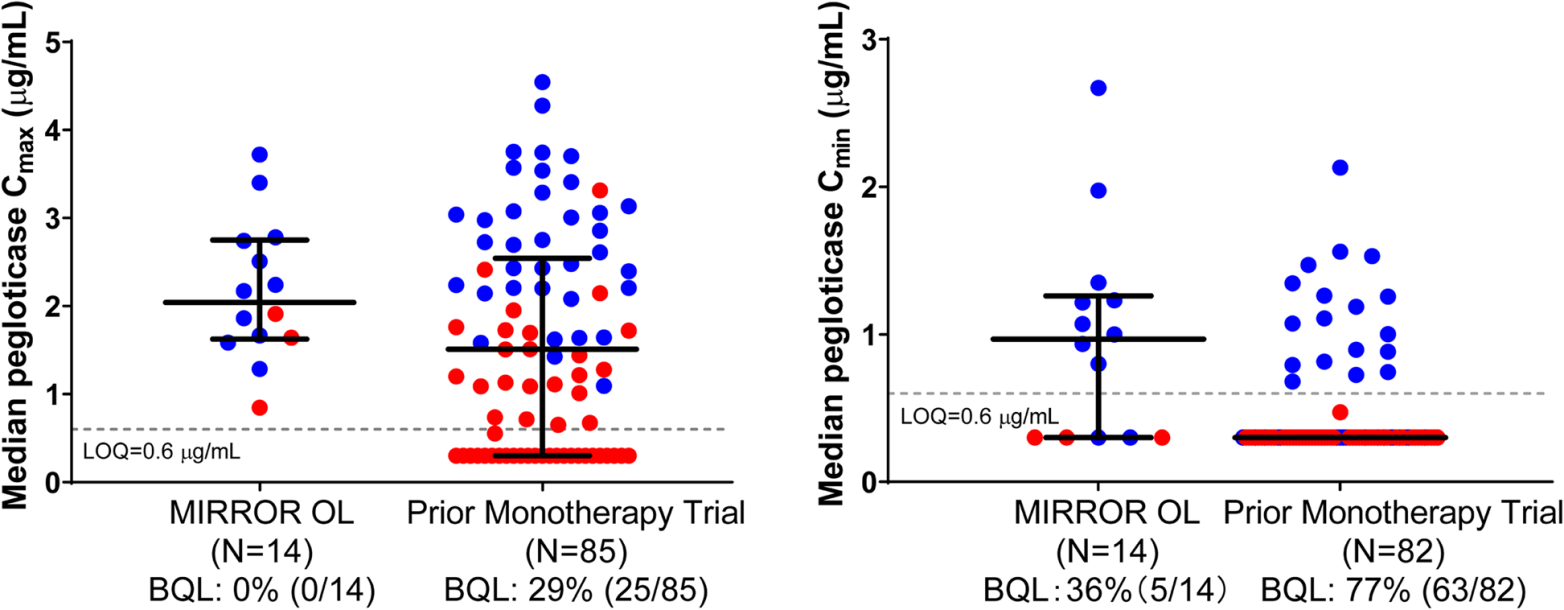
Comparison of pegloticase exposure with methotrexate co-treatment in the current study (MIRROR OL) and as monotherapy in prior phase 3 trials. Blue circles represent responders, and red circles represent non-responders. The gray dotted line shows the limit of quantitation (LOQ) of pegloticase measurements (0.6 µg/mL). Data below LOQ (BQL) were imputed as 0.3 µg/mL

**Fig. 3:**
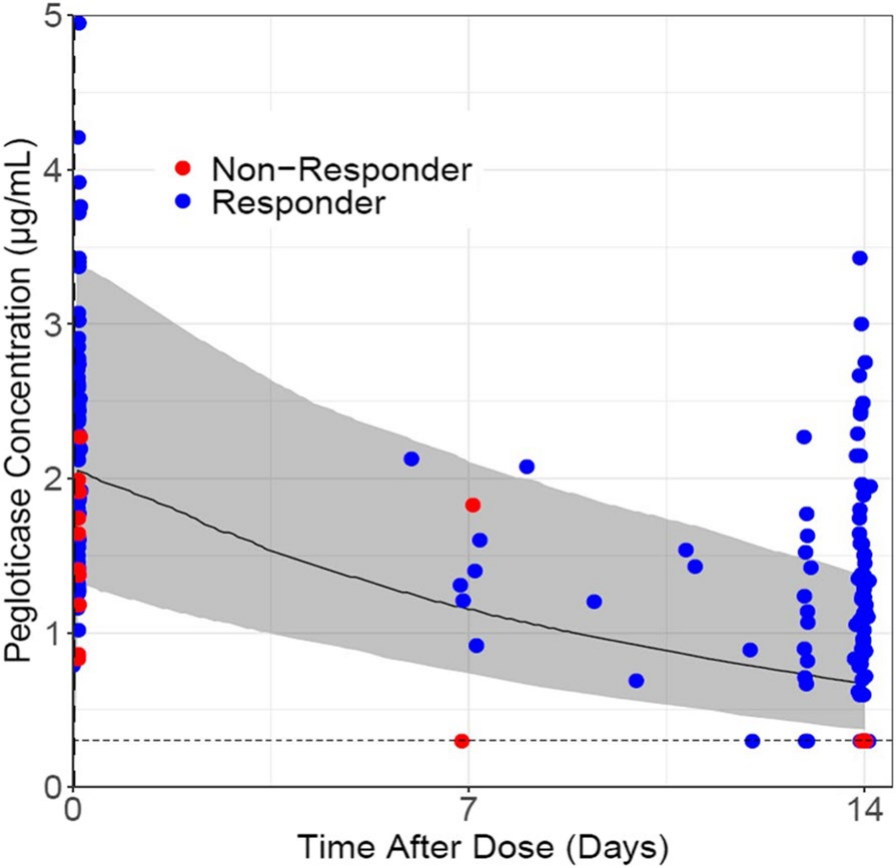
Comparison of observed pegloticase concentrations in the current study (MIRROR OL, pegloticase + methotrexate co-therapy) and in a simulated PK profile of prior phase 3 trials (pegloticase monotherapy). Circles represent the observed data in MIRROR OL with non-responders in red and responders in blue. Simulated monotherapy pegloticase concentration over time is shown as the median concentration (black line) with 90% confidence intervals (gray-shaded area). The simulation was modeled using the time elapsed from the start of each infusion, pooling data from all 12 infusions administered to phase 3 pivotal trial participants. Values below the limit of quantitation were imputed as 0.3 μg/ml (dotted line)

The measured concentrations of MTX-PGs were maintained during the treatment course, suggesting compliance with MTX administration [see Additional file 3]. Concomitant treatment with methotrexate generally improved pegloticase exposures, resulting in a lower proportion of patients with *C*_min_ BQL on methotrexate (5/14 [36%] vs. without methotrexate 63/82 [77%]) and higher overall *C*_min_ (median [Q1, Q3] 1.03 (BQL, 1.23) µg/mL with methotrexate vs. BQL [BQL, BQL] without methotrexate), as well as slightly higher peak concentration *C*_max_ (median [Q1, Q3] 2.11 [1.65, 2.59] µg/mL with methotrexate vs. 1.51 [BQL, 2.48] µg/mL without methotrexate; Fig. 2, Table 3). Consistently, pegloticase + methotrexate co-treatment resulted in more observed pegloticase concentrations above the predicted median value of pegloticase monotherapy (66% for the entire 14-day dosing interval and 82% for trough concentrations, Fig. 3). All non-responders had pegloticase *C*_min_ BQL across time points, whereas responders typically had measurable *C*_min_ values across most time points with the exception of two patients that only have *C*_min_ above BQL for a few visits (Fig. 2).

**Table 3:** Summary of pegloticase exposure with methotrexate co-treatment (current study) and as monotherapy (calculated using pharmacokinetic findings of prior phase 3 trials [23, 26])

	***C***_**max**_** (µg/mL)**		***C***_**min**_** (µg/mL)**	
	Pegloticase + MTX	Pegloticase (monotherapy trial)	Pegloticase + MTX	Pegloticase (monotherapy trial)
Median (Q1, Q3)	2.11 (1.65, 2.59)	1.51 (BQL, 2.48)	1.03 (BQL, 1.23)	BQL (BQL, BQL)
BQL, *n*/*N* (%)	0/14 (0%)	25/85 (29%)	5/14 (36%)	63/82 (77%)

*C*_max_ Maximum pegloticase concentration during the treatment period, *C*_min_ Minimum pegloticase concentration during the treatment period, *BQL* Below quantitation limit (0.6 µg/mL), *MTX* Methotrexate

### Immunogenicity

ADA data are consistent with the improved pegloticase efficacy and PK when co-administered with methotrexate. An increase in ADA titer was observed in 2 of 3 non-responders (1 patient from < 10 at baseline to 320 at week 4, 1 patient 40 at baseline to 640 at week 2) and temporally corresponded with the loss of pegloticase exposure and SU increase. The third non-responder received 5 pegloticase infusions prior to meeting the SU discontinuation criteria. This patient was positive for anti-PEG antibody titer prior to the first pegloticase dose but negative during the treatment period. Two of eleven responders at month 6 showed a small increase (≤ 40) in anti-PEG antibody titer at a single time during pegloticase + methotrexate treatment. All other anti-PEG antibody titer measurements were either negative or no greater than the baseline levels. The remaining 9 responders were considered negative for the induction of anti-PEG antibodies.

### Safety

Ten patients (66.7%) experienced an AE during the run-in period, including gout flare (5 patients), nausea (2 patients), and abdominal discomfort (2 patients, Table 4). All patients experienced one or more AEs during pegloticase + methotrexate Treatment, most commonly gout flare (13 patients [92.9%]). Patients also experienced diarrhea, nasopharyngitis, upper respiratory tract infection, muscle strain, and arthralgia (3 patients each [21.4%], Table 4). One SAE of bacterial sepsis secondary to cholecystitis was observed and deemed unrelated to the study treatments by the reporting investigator.

**Table 4:** Summary of adverse events and serious adverse events

	**MTX run-in** (4 weeks), ITT population (*N* = 15)	**Pegloticase + MTX treatment** (up to 52 weeks), mITT population (*N* = 14)
Any AE, *n* (%)	10 (66.7%)	14 (100%)
Any SAE, *n* (%)	0 (0.0%)	1 (7.1%)^a^
AEs occurring in > 1 patient in either period, *n* (%)		
Gout flare	5 (33.3%)	13 (92.9%)
Diarrhea	1 (6.7%)	3 (21.4%)
Nasopharyngitis	1 (6.7%)	3 (21.4%)
Upper respiratory tract infection	0 (0.0%)	3 (21.4%)
Muscle strain	0 (0.0%)	3 (21.4%)
Arthralgia	0 (0.0%)	3 (21.4%)
Sinusitis	0 (0.0%)	2 (14.3%)
Hypertension	0 (0.0%)	2 (14.3%)
Liver function test values increased	0 (0.0%)	2 (14.3%)^b^
Nausea	2 (13.3%)	1 (7.1%)
Abdominal discomfort	2 (13.3%)	0 (0.0%)
AEs of special interest, *n* (%)		
Infusion reactions	–	1 (7.1%)^c^
Anaphylaxis	–	0 (0.0%)
Cardiovascular events	0 (0.0%)	0 (0.0%)
Gout flare (patients with ≥ 1 flare)	5 (33.3%)	13 (92.9%)
Number of flares, mean ± SD [range]	1.2 ± 0.5 [1, 2]	6.5 ± 5.8 [1–20]
Number of flares, median	1	5

*MTX* Methotrexate, *ITT* Intent-to-treat (any patient exposed to MTX during the run-in period), *mITT* Modified intent-to-treat (any patient exposed to pegloticase during the pegloticase + MTX treatment period)

^a^ Bacterial sepsis unrelated to the study treatment; patient remained in the study on treatment

^b^ One patient had a grade 1 ALT increase (82 U/L), and 1 patient had a grade 1 increase in both ALT (80 U/L) and AST (53 U/L); both patients had AE resolution following a methotrexate dose reduction to 10 mg/week

^c^ Mild cough after infusion 5; patient remained on therapy, completed the study at week 24, and was a treatment responder during month 6

**Fig. 4:**
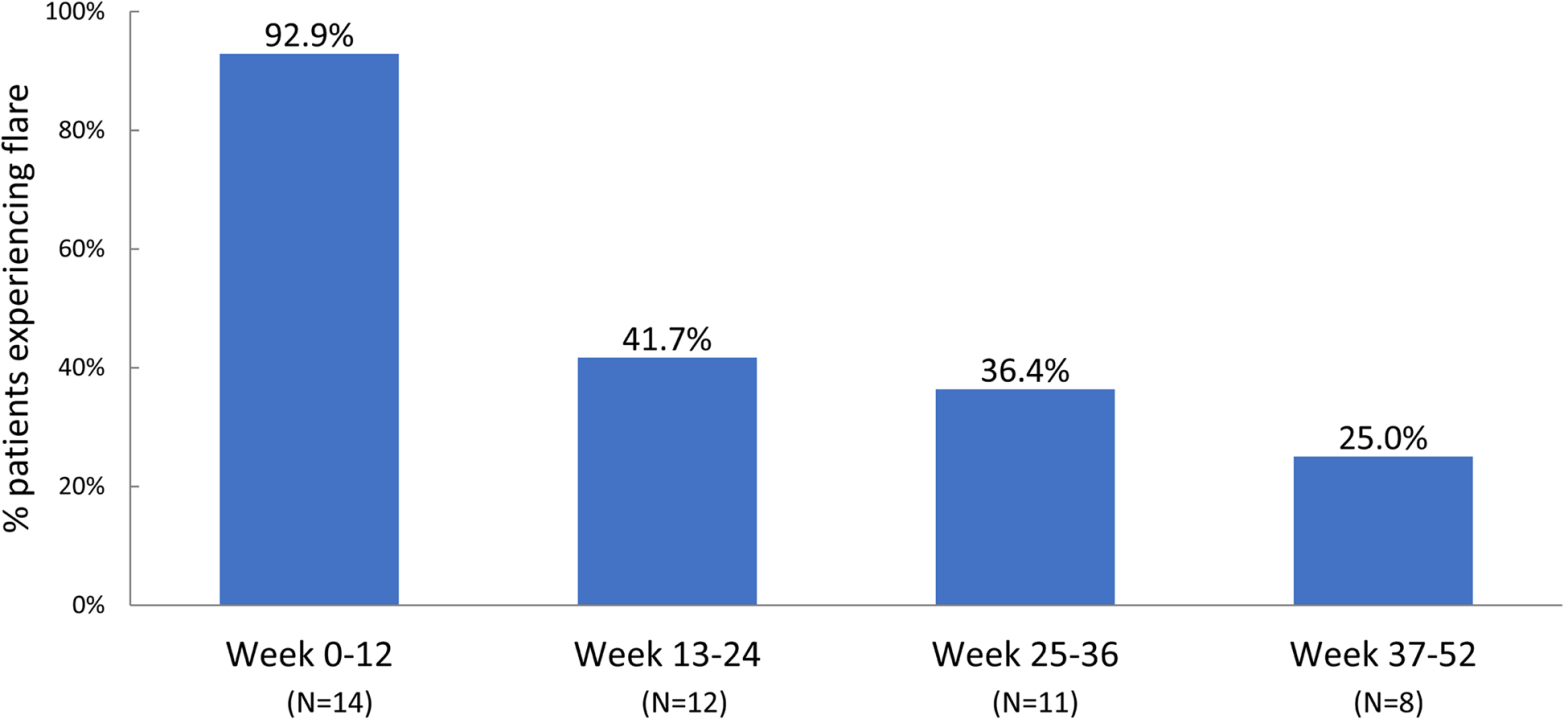
Proportion of patients on treatment experiencing gout flares during the pegloticase + methotrexate treatment period. The mean number of flares during weeks 0–12 and weeks 37–52 was 4.2 ± 2.3 (range 1 − 8) and 2.5 ± 0.7 (range 2 − 3), respectively

Infusion reaction, anaphylaxis, cardiovascular events, and gout flare were AEs of special interest. A single IR was reported in one patient following the 5th pegloticase infusion (week 8 visit). As previously detailed [33], the event was described by the investigator as a mild cough that began towards the end of the infusion and resolved without treatment after approximately 1 h. Typical IR signs (hives, itching, shortness of breath, sweating, chills) were not present. In this patient, SU remained BQL, and the patient met the responder criteria during month 6 (exited study at week 24). Anaphylaxis, cardiovascular event, and major adverse cardiovascular event (including non-fatal myocardial infarction, non-fatal stroke, cardiovascular death, and congestive heart failure) were not observed during any study period. Thirteen patients (92.9%) experienced gout flares during treatment (2 severe flares). However, both the number of patients on treatment who flared and the frequency of flares decreased over time, from 4.2 ± 2.3 flares (range 1–8 flares) in 13 of 14 patients (92.9%) during the initial 12 weeks of treatment to 2.5 ± 0.7 flares (range 2–3 flares) in 2 of 8 patients (25.0%) during weeks 37–52 (Fig. 4).

**Fig. 5:**
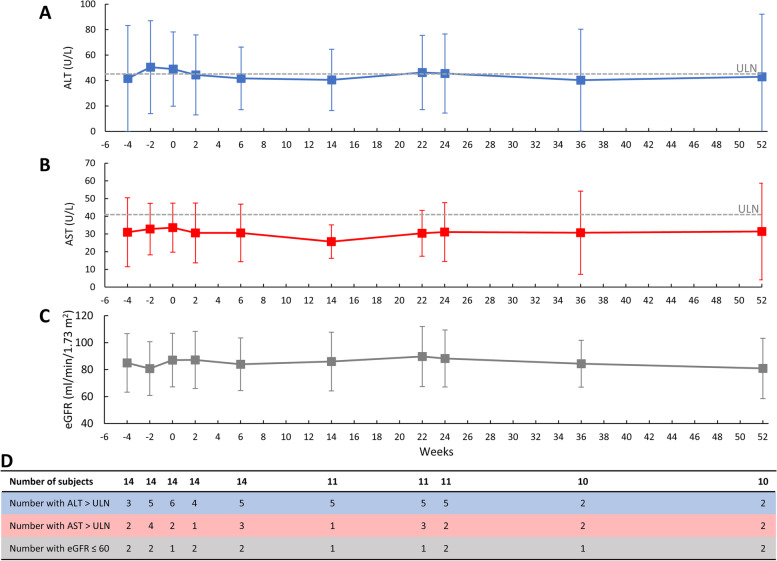
Liver **a**, **b** and renal function **c** test results through the run-in (weeks − 4 to 0) and treatment (weeks 0 to 52) periods. Week − 4 values were measured prior to methotrexate exposure. Week 0 (day 1) values were measured prior to pegloticase exposure. The number of patients with liver function tests above the upper limits of normal and estimated glomerular filtration rate < 60 mL/min/1.73 m.^2^ are also shown **d**. Error bars represent the standard deviation. ALT, alanine aminotransferase; AST, aspartate aminotransferase; eGFR, estimated glomerular filtration rate (calculated from serum creatinine measurements using the MDRD equation)

Liver and kidney functions were carefully monitored during both the run-in and treatment periods. At screening, 3 patients in the mITT population (21.4%) had an ALT above the upper limit of normal (ULN), and 2 patients (14.3%) had an AST above the ULN. A minor, transient increase in LFTs occurred shortly after methotrexate initiation, with LFTs stabilizing below the ULN at week 2 (Fig. 5a, b). LFTs remained stable through week 52. Two patients experienced one mild (grade 1) LFT elevation, both of which resolved following a methotrexate dose reduction (15 to 10 mg/week at week 25 in 1 patient [titrated back to 15 mg/week beginning at week 37] and week 2 in 1 patient). Methotrexate was otherwise well-tolerated in the mITT population. One patient had an accidental dose reduction to 12.5 mg/week on 2 occasions (weeks 9 and 18). All 3 patients who had a methotrexate dose reduction remained treatment responders during the pegloticase + methotrexate treatment period.

The mean estimated glomerular filtration rate (eGFR) was 84.6 ± 21.7 mL/min/1.73 m^2^ just prior to beginning methotrexate (week − 4, *n* = 14) and remained stable throughout the methotrexate run-in and pegloticase + methotrexate treatment periods (Fig. 5c). The mean eGFR was 88.3 ± 21.1 mL/min/1.73 m^2^ at week 24 (change from week − 4, + 4.2 ± 14.9 mL/min/1.73 m^2^, *n* = 11) and 80.9 ± 22.4 mL/min/1.73 m^2^ at week 52 (change from week − 4, − 4.0 ± 18.3 mL/min/1.73 m^2^, *n* = 10).

## Discussion

Compared to previously reported responder rates for pegloticase monotherapy, an increased proportion of patients treated with methotrexate in conjunction with pegloticase maintained therapeutic response during month 6 (42% vs. 79% [24]). Furthermore, all 6-month responders who remained on pegloticase + methotrexate co-therapy continued to be treatment responders through month 12 (SU < 1 mg/dL in all 8 patients). The duration of treatment response to pegloticase is of vital importance to patients with uncontrolled gout, many of whom pegloticase is their last medical treatment option.

The increased pegloticase treatment response rate is almost certainly attributable to the addition of methotrexate. Pegloticase is highly effective in lowering SU, but many patients have an incomplete response due to developing ADAs targeting polyethylene glycol moieties resulting in increased pegloticase clearance [24, 27]. When compared to historical phase 3 pivotal trial data, PK and ADA data from the current trial suggest that methotrexate reduces the immunogenicity of pegloticase and prolongs the biologic activity of pegloticase. Similar findings have been previously observed with other biologics (e.g., infliximab [39], adalimumab [40, 41], and certolizumab pegol [42]). The median pegloticase *C*_min_ was higher (1.03 μg/mL with methotrexate vs. BQL without methotrexate), and the proportion of patients with a pegloticase *C*_min_ BQL (36% vs. 77%) was lower in patients treated with pegloticase + methotrexate co-therapy than those treated with pegloticase monotherapy (the lowest quantitation limit was similar between studies). Furthermore, 2 of 11 primary endpoint responders in the current trial developed low titer (≤ 40) anti-PEG antibodies at a single visit following pegloticase treatment (the remaining 9 responders were ADA-negative or did not exceed their baseline ADA levels). In contrast, 2 of the 3 non-responders developed ADA titers concomitantly with an undetectable serum pegloticase concentration and increase in SU above 6 mg/dL. The remaining non-responder was positive for anti-PEG antibody titer prior to the first pegloticase dose, but negative thereafter. Pre-infusion serum pegloticase concentration was undetectable throughout treatment for reasons that remain unclear. It is worth noting that the detection of antibody formation is dependent on assay sensitivity and specificity and that the incidence of antibody positivity may be influenced by assay processing methodology. Therefore, a comparison of antibody incidence in the current study to historical pegloticase studies may not be appropriate. Concentrations of MTX-PGs were maintained during the treatment course, suggesting compliance with methotrexate administration. Additionally, there was no apparent difference in MTX-PG concentrations between responders and non-responders (data not shown).

Extended safety data indicate that pegloticase + methotrexate co-therapy was well tolerated over the 52-week treatment period, and no new safety concerns over pegloticase monotherapy or the 6-month findings [34] were identified. Gout flares remained the most common AE observed (13 of 14 patients [92.9%]) and occurred in most patients during the first 12 weeks of pegloticase therapy. However, both the number of patients experiencing flares and the frequency of flares markedly decreased over time. On average, worsening of hepatic or renal function was not noted throughout the run-in or treatment periods. Two AEs of increased LFTs were observed. Both were graded as mild and resolved with methotrexate dose reduction. Other AEs that were observed in more than 1 patient during the treatment period included diarrhea, nasopharyngitis, upper respiratory tract infection, arthralgia, muscle strain, sinusitis, and hypertension.

This study was limited by its small size, open-label design, and lack of a control group. Larger, randomized, placebo-controlled trials are needed to more rigorously examine the benefits and associated risks of administering methotrexate in conjunction with pegloticase to patients with uncontrolled gout. Such a trial has recently been completed (MIRROR RCT, NCT03994731). Our study findings prompt several clinical questions that cannot be answered with these data. First, it is not unheard of for patients to be treated with pegloticase for longer than 12 months. However, this study did not examine the risks and benefits of pegloticase + methotrexate co-therapy past treatment month 12. Second, the question of when to re-initiate oral ULT following pegloticase discontinuation remains. Some physicians restart oral ULT immediately in tolerant patients, while others wait until SU rises above 6 mg/dL. Lastly, there remains the question if lower doses of methotrexate may be able to achieve similar successful results as those seen here. Further studies to answer all of these questions are needed.

## Conclusions

In conclusion, these data further support the use of methotrexate with pegloticase in patients with uncontrolled gout. New pharmacokinetic and ADA data presented here strongly suggest that methotrexate reduces immunogenicity to pegloticase, allowing more patients to accomplish treatment goals from full courses of therapy with a reduction in previously seen adverse effects. This benefit seems to be sustained for at least 12 months in patients remaining on co-therapy with both pegloticase and methotrexate.

## Supplementary Information

**Additional file 1: Supplement Table 1a.** Schedule of assessments, screening through Week 24. **Supplement Table 1b.** Schedule of assessments, Weeks 26-52.

**Additional file 2: Supplemental Figure 1.** Serum urate levels in the two patients treated with pegloticase+methotrexate co-therapy for 24 weeks. Both patients met pegloticase treatment goals and continued in study on observation. Allopurinol was started at the investigators’ discretion. One patient (Patient 1) initiated allopurinol at Week 24 (300 mg/day for 1 week [Week 25], 600 mg for 2 weeks [Week 27], 300 mg/day thru Week 52) and the other (Patient 2) initiated allopurinol 2 weeks after discontinuing pegloticase+methotrexate (150 mg/day until Week 47, 300 mg/day thru Week 52).

**Additional file 3: Supplemental Figure 2.** Individual concentration-time course of pegloticase and MTX polyglutamates.

## Data Availability

Horizon is committed to responsibly sharing data from the clinical trials we sponsor. Access to anonymized, individual, and trial-level data (analysis data sets) may be granted to qualified researchers for independent scientific research, provided the trials are not part of an ongoing or planned regulatory submission (including clinical trial data for unlicensed products and indications). Data may be requested by submitting a research proposal and statistical analysis plan and will be provided following review and approval of the plan and execution of a data sharing agreement. For more information, or to submit a request, please submit to medicalinformation@horizontherapeutics.com.

## Abbreviations

SU: Serum urate
ULT: Urate-lowering therapy
IR: Infusion reaction
ADA: Anti-drug antibody
BQL: Below quantification limits
