# Supplementary material for: A multicentre, efficacy and safety study of methotrexate to increase response rates in patients with uncontrolled gout receiving pegloticase (MIRROR): 12-month efficacy, safety, immunogenicity, and pharmacokinetic findings during long-term extension of an open-label study
Source: Arthritis Res Ther. 2022 Aug 25;24:208. doi: 10.1186/s13075-022-02865-z (PMC9404640; doi:10.1186/s13075-022-02865-z)
Supplement: Supplementary file 1 — Additional file 1: Supplement Table 1a. Schedule of assessments, screening through Week 24. Supplement Table 1b. Schedule of assessments, Weeks 26-52. [file 13075_2022_2865_MOESM1_ESM.pdf]

Supplement Table 1a. Schedule of assessments, screening thru Week 24.

	**Screen**[1]	**MTX Run-in**[2] (4 weeks)		**Pegloticase + MTX Treatment**[3] (Day 1 through Week 24)														
		**-4 W**	**-2 W**	**D1**	**W1**	**W2**	**W4**	**W6**	**W7**	**W8**	**W10**	**W12**	**W14**	**W16**	**W18**	**W20**	**W22**	**W24**
**Study Procedure/Assessment**				**Inf 1**		**Inf 2**	**Inf 3**	**Inf 4**		**Inf 5**	**Inf 6**	**Inf 7**	**Inf 8**	**Inf 9**	**Inf 10**	**Inf 11**	**Inf 12**	
Informed consent	X																	
Enrollment				X														
Demographic data	X																	
Inclusion/exclusion criteria	X	X	X	X														
Medical/surgical history[4]	X	X																
Medication/substance use history[5]	X	X	X															
Physical examination[6]	X	X		X			X			X		X		X		X		X
Vital signs, height, weight[7]	X	X		X		X	X	X		X	X	X	X	X	X	X	X	X
Electrocardiogram[8]				X														
HIV antibody screening	X																	
AS/SAE assessment[9]	X	X	X	X	X	X	X	X	X	X	X	X	X	X	X	X	X	X
Concomitant medications				X	X	X	X	X	X	X	X	X	X	X	X	X	X	X
Gout flare assessment	X	X	X	X		X	X	X		X	X	X	X	X	X	X	X	X
Swollen and tender joint counts		X		X									X					X
HAQ	X	X		X									X					X
Patient global assessment	X	X		X									X					X
Physician global assessment	X	X		X									X					X
Joint pain assessment	X	X		X									X					X
DECT[10]				X														
Tophi assessment	X																	X
MTX dosing calendar		X	X	X	X	X	X	X	X	X	X	X	X	X	X	X	X	X
MTX dispensed[11]		X	X	X		X	X	X		X	X	X	X	X	X	X	X	X
MTX compliance/reconciliation			X	X		X	X	X		X	X	X	X	X	X	X	X	X
IR prophylaxis compliance[12]				X		X	X	X		X	X	X	X	X	X	X	X	X
FA/GF prophylaxis compliance[13]			X	X		X	X	X		X	X	X	X	X	X	X	X	X
Pre-infusion MTX polyGL sampling[14]				X			X			X							X	X
Pegloticase PK sampling[15]				X	X	X	X	X	X	X	X		X		X		X	X

	Screen[1]	MTX Run-in[2] (4 weeks)		Pegloticase + MTX Treatment[3] (Day 1 through Week 24)														
		W-4	W-2	D1	W1	W2	W4	W6	W7	W8	W10	W12	W14	W16	W18	W20	W22	W24
**Study Procedure/Assessment**				Inf 1		Inf 2	Inf 3	Inf 4		Inf 5	Inf 6	Inf 7	Inf 8	Inf 9	Inf 10	Inf 11	Inf 12	
SU[16]	X	X	X	X	X	X	X	X	X	X	X	X	X	X	X	X	X	X
Hematology	X	X	X	X		X		X					X				X	X
Clinical chemistry	X	X	X	X		X		X					X				X	X
Spot urine collection	X	X	X	X		X		X					X				X	X
Antibody sample[17]				X	X	X	X	X	X	X	X		X		X		X	X
G6PD deficiency screening	X																	
Urine pregnancy test[18]	X	X	X	X		X	X	X		X	X	X	X	X	X	X	X	X
Partner pregnancy inquiry[19]																		
Clinical status assessment[20]																		X

MTX, methotrexate; D, day; W, week; Inf, infusion; AE, adverse event; SAE, serious adverse event; HAQ, health assessment questionnaire; DECT, dual-energy computed tomography; IR, infusion reaction; FA/GF, Folic acid/gout flare; polyGL, poly glutamate; PK, pharmacokinetic; SU, serum urate.

[1]Screening included the MTX Run-in Period and the Screening visit occurred up to 2 weeks prior to the MTX Run-in period.
[2]Subjects took 15 mg oral MTX per week during the 4-week Run-in Period.
[3]Subjects took 15 mg oral MTX per week and received 8 mg intravenous pegloticase every 2 weeks (total of 12 infusions).
[4]The Investigator (or their designee) collected a complete gout history and other relevant medical/surgical history.
[5]Included gout medication history (from diagnosis through MTX Run-in Period), substance use history, and non-gout medications used within a year of Screening.
[6]Clinically significant findings were recorded as AEs. Assessment of tophi was conducted at Screening and Week 24.
[7]Heart rate and blood pressure was measured after subject had been sitting and calm/rested for ≥5 minutes. Recorded vital signs were not measured during pegloticase infusion. Weight was measured without shoes on Day 1 and at Weeks 8, 16, and 24. Height was measured only at the Screening visit.
[8]Electrocardiograms were recorded prior to the first pegloticase infusion.
[9]Investigators completed additional information for possible infusion reactions or anaphylaxis.
[10]At sites with DECT capabilities, DECT images were obtained at Day 1 and Week 24.
[11]MTX was dispensed and brought back to each visit to check compliance. If the subject required an MTX dose reduction, the Investigator changed the number of tablets to take weekly. The updated number of tablets, along with the date and time of each MTX dose, was recorded in the dosing calendar. MTX should have been taken 1 to 3 days prior to each pegloticase infusion, but was required ≥60 min prior to each infusion.

[12]Infusion prophylaxis consisted of oral fexofenadine (60 or 180 mg based on Investigator's discretion) the day before and morning of infusion, oral acetaminophen (1000mg) the morning of infusion, and intravenous methylprednisolone (125 mg infused over 10-3- minutes) or hydrocortisone (200 mg) immediately prior to each infusion.

[13] Subjects were required to complete at least one standard gout flare prophylaxis protocol (e.g., colchicine and/or non-steroidal anti=inflammatory drugs and/or low-dose prednisone ≤10 mg/day) at least 1 week before the first pegloticase dose and continue flare prophylaxis per American College of Rheumatology guidelines (the longer of 6 months or 3 months after first SU reading ≤6 mg/dL [6 months after SU reading ≤5 mg/dL for patients with tophaceous gout]). Subjects took 1 mg oral folic acid every day from Week -4 to the end of the study.

[14]Blood samples were collected prior to pegloticase infusion and after the end of infusion on Day 1 and at Weeks 4, 8, 22, and 24 to measure MTX polyglutamate levels.

[15]Blood samples were collected prior to pegloticase infusion and after the end of infusion on Day 1 and at Weeks 2, 4, 6, and 8 and prior to pegloticase infusion at Weeks 10, 14, 18, 22, and 24. Randomly selected subjects, who consented for frequent PK sampling, also had PK sampling at Weeks 1 and 7 (morning preferred).

[16]Serum samples for SU levels were collected at Screening (≤2 weeks of first MTX dose at Week -4), Week -4 (prior to first MTX dose), Week -2, and within 48 hours prior to and immediately after the end of each pegloticase infusion (except on Day 1). Randomly selected subjects who consented to more frequent sampling also had SU levels measured at Weeks 1 and 7. Two blood samples were collected for SU level measurement; one sample was tested by the site's local laboratory and the other was sent to the central laboratory. A subject with an SU level ≥6 mg/dL at 2 consecutive visits, beginning at Week 2, was classified as a non-responder. The subjects continued in the study without further pegloticase/methotrexate therapy.

[17]Serum samples for evaluation of anti-PEG and anti-uricase IgG antibodies were collected prior to pegloticase infusion on Day 1 and at Weeks 2, 4, 6, 8, 10, 14, 18, 22, and 24. Visits for frequent sampling of a subset of subjects who consented for additional non-infusion visit PK sampling (random, morning preferred) also had antibody evaluation at Weeks 1 and 7. In the event of a suspected infusion reaction, a serum sample was collected at that time or at the subsequent visit for evaluation of pegloticase antibodies.

[18]For women of childbearing potential, a serum pregnancy test was performed at Screening. A urine pregnancy test was performed at each visit (except Weeks 1 and 7 in high frequency PK sampling subjects) thereafter.

[19]Non-vasectomized males were asked about partner pregnancy.

[20]The Investigator reviewed the clinical status of the subject at Week 24.

Supplement Table 1b. Schedule of assessments, Weeks 26-52.

	Pegloticase + MTX Treatment[1] (Week 26 through Week 50)													End of infusions	EOS/ ET	Safety Contact	MTX partner pregnancy follow-up	Follow -up
	**W26**	**W28**	**W30**	**W32**	**W34**	**W36**	**W38**	**W40**	**W42**	**W44**	**W46**	**W48**	**W50**	**≤2 wks of final inf**	**Wk 52**	**30 days after last inf**	**3 mo after last MTX**	**3 and 6 mo**
**Study Procedure/Assessment**	**Inf 14**	**Inf 15**	**Inf 16**	**Inf 17**	**Inf 18**	**Inf 19**	**Inf 20**	**Inf 21**	**Inf 22**	**Inf 23**	**Inf 24**	**Inf 25**	**Inf 26**					
Physical examination[2]						X								X	X			X
Vital signs, height, weight[3]	X	X	X	X	X	X	X	X	X	X	X	X	X	X	X			X
AS/SAE assessment[4]	X	X	X	X	X	X	X	X	X	X	X	X	X	X	X	X		X
Concomitant medications	X	X	X	X	X	X	X	X	X	X	X	X	X	X	X			X
Gout flare assessment	X	X	X	X	X	X	X	X	X	X	X	X	X	X	X			X
Swollen/tender joint counts						X								X	X			X
HAQ						X								X	X			X
Patient global assessment						X								X	X			X
Physician global assessment						X								X	X			X
Joint pain assessment						X								X	X			X
DECT[5]						X								X	X			X
Tophi assessment						X								X	X			X
MTX dosing calendar	X	X	X	X	X	X	X	X	X	X	X	X	X					
MTX dispensed[6]	X	X	X	X	X	X	X	X	X	X	X	X	X					
MTX compliance/reconciliation	X	X	X	X	X	X	X	X	X	X	X	X	X	X	X			
IR prophylaxis compliance[7]	X	X	X	X	X	X	X	X	X	X	X	X	X					
FA/GF prophylaxis compliance[8]	X	X	X	X	X	X	X	X	X	X	X	X	X	X	X			
Pegloticase infusion	X	X	X	X	X	X	X	X	X	X	X	X	X					
Pre-infusion MTX polyGL sampling[9]						X												
Pegloticase PK sampling[10]						X								X	X			
SU[11]	X	X	X	X	X	X	X	X	X	X	X	X	X	X	X			X
Hematology						X								X	X			X
Clinical chemistry						X								X	X			X
Spot urine collection						X								X	X			

Antibody sample[12]						X								X	X			X
Urine pregnancy test[13]	X	X	X	X	X	X	X	X	X	X	X	X	X	X	X	X		
Partner pregnancy inquiry[14]																	X	
Clinical status assessment[15]														X	X			

MTX, methotrexate; EOS/ET, end of study/early termination; D, day; W, week; mo, month; Inf, infusion; AE, adverse event; SAE, serious adverse event; HAQ, health assessment questionnaire; DECT, dual-energy computed tomography; IR, infusion reaction; FA/GF, Folic acid/gout flare; polyGL, poly glutamate; PK, pharmacokinetic; SU, serum urate.

[1]Subjects took 15 mg oral MTX per week and received 8 mg intravenous pegloticase every 2 weeks (total of 12 infusions Weeks).

[2]Clinically significant findings were recorded as AEs. Assessment of tophi was conducted at Week 36, the End of Pegloticase Treatment, Week 52 (or End of Study/Early Termination), and Post Treatment 3 and 6 month Follow-up visits.

[3]Heart rate and blood pressure were measured after subject had been sitting and calm/rested for ≥5 minutes. Recorded vital signs were not measured during pegloticase infusion. Weight was measured without shoes at Week 36, the End of Pegloticase Treatment, Week 52 (or End of Study/Early Termination), and Post Treatment 3 and 6 month Follow-up visits. Height was measured only at the Screening visit.

[4]Investigators completed additional information for possible infusion reactions or anaphylaxis.

[5]At sites with DECT capabilities, DECT images were obtained at Week 36, the End of Pegloticase Treatment, Week 52 (or End of Study/Early Termination), and Post Treatment 3 and 6 month Follow-up visits.

[6]MTX was dispensed and brought back to each visit to check compliance. If the subject required an MTX dose reduction, the Investigator changed the number of tablets to take weekly. The updated number of tablets, along with the date and time of each MTX dose, was recorded in the dosing calendar. MTX should have been taken 1 to 3 days prior to each pegloticase infusion, but was required ≥60 min prior to each infusion.

[7]Infusion prophylaxis consisted of oral fexofenadine (60 or 180 mg based on Investigator's discretion) the day before and morning of infusion, oral acetaminophen (1000mg) the morning of infusion, and intravenous methylprednisolone (125 mg infused over 10-3- minutes) or hydrocortisone (200 mg) immediately prior to each infusion.

[8]Subjects were required to complete at least one standard gout flare prophylaxis protocol (e.g., colchicine and/or non-steroidal anti=inflammatory drugs and/or low-dose prednisone ≤10 mg/day) at least 1 week before the first pegloticase dose and continue flare prophylaxis per American College of Rheumatology guidelines (the longer of 6 months or 3 months after first SU reading ≤6 mg/dL [6 months after SU reading ≤5 mg/dL for patients with tophaceous gout]). Subjects took 1 mg oral folic acid every day from Week -4 to the end of the study.

[9]Blood samples were collected prior to pegloticase infusion and after the end of infusion at Week 36 to measure MTX polyglutamate levels.

[10]Blood samples were collected prior to pegloticase infusion and after the end of infusion on Day 1 and at Weeks 2, 4, 6, and 8 and prior to pegloticase infusion at Week 36, the End of Pegloticase Treatment, and Week 52 (or End of Study/Early Termination).

[11]Serum samples for SU levels were collected at within 48 hours prior to and immediately after the end of each pegloticase infusion, at the End of Pegloticase Treatment, at Week 52 (or End of Study/Early Termination), and Post Treatment 3 and 6 month Follow-up visits. Two blood samples were collected for SU level measurement; one sample was tested by the site's local laboratory and the other was sent to the central laboratory. A subject with an SU level ≥6 mg/dL at 2 consecutive visits, beginning at Week 2, was classified as a non-responder. The subjects continued in the study without further pegloticase/methotrexate therapy.

[12]Serum samples for evaluation of anti-PEG and anti-uricase IgG antibodies were collected prior to pegloticase infusion at Week 36 and the End of Pegloticase Treatment. Samples were also collected at Week 52 (or End of Study/Early Termination) and Post Treatment 3 and 6 month Follow-up visits. In the event of a suspected infusion reaction, a serum sample was collected at that time or at the subsequent visit for evaluation of pegloticase antibodies.

[13]For women of childbearing potential, a urine pregnancy test was performed at each visit.

[14] Subjects who were non-vasectomized males were asked 3 months after MTX discontinuation regarding partner pregnancy. This occurred at a regulatory scheduled visit or by a separate phone/email/site visit.

[15]The Investigator reviewed the clinical status of the subject at End of Pegloticase Treatment and Week 52 (or End of Study/Early Termination).
