# Supplementary material for: A multicentre, efficacy and safety study of methotrexate to increase response rates in patients with uncontrolled gout receiving pegloticase (MIRROR): 12-month efficacy, safety, immunogenicity, and pharmacokinetic findings during long-term extension of an open-label study
Source: Arthritis Res Ther. 2022 Aug 25;24:208. doi: 10.1186/s13075-022-02865-z (PMC9404640; doi:10.1186/s13075-022-02865-z)
Supplement: Supplementary file 2 — Additional file 2: Supplemental Figure 1. Serum urate levels in the two patients treated with pegloticase+methotrexate co-therapy for 24 weeks. Both patients met pegloticase treatment goals and continued in study on observation. Allopurinol was started at the investigators’ discretion. One patient (Patient 1) initiated allopurinol at Week 24 (300 mg/day for 1 week [Week 25], 600 mg for 2 weeks [Week 27], 300 mg/day thru Week 52) and the other (Patient 2) initiated allopurinol 2 weeks after discontinuing pegloticase+methotrexate (150 mg/day until Week 47, 300 mg/day thru Week 52). [file 13075_2022_2865_MOESM2_ESM.pdf]

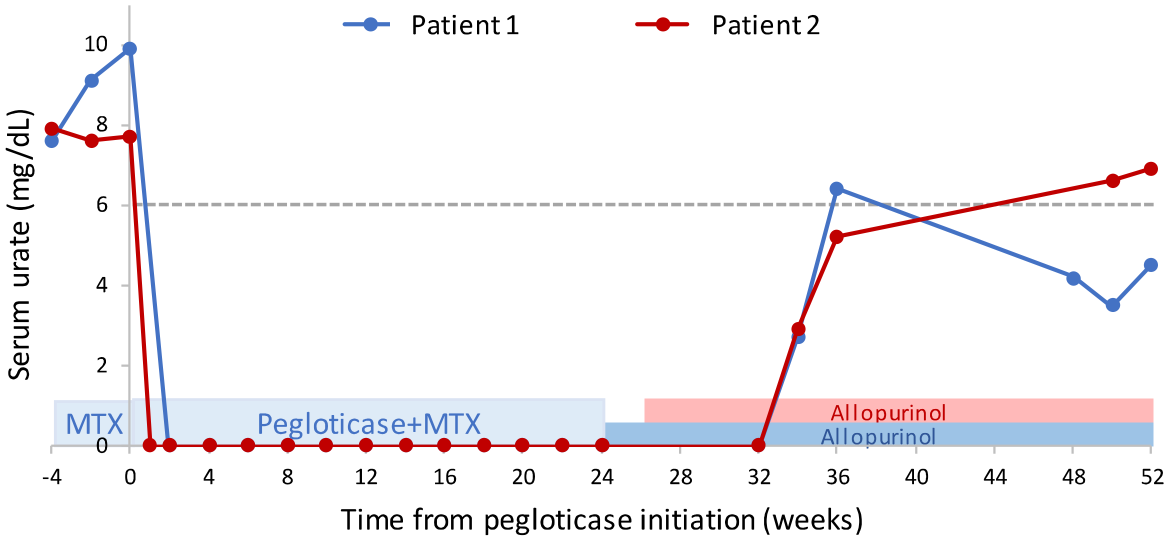

Supplemental Figure 1. Serum urate levels in the two patients treated with pegloticase+methotrexate co-therapy for 24 weeks. Both patients met pegloticase treatment goals and continued in study on observation. Allopurinol was started at the investigators' discretion. One patient (Patient 1) initiated allopurinol at Week 24 (300 mg/day for 1 week [Week 25], 600 mg for 2 weeks [Week 27], 300 mg/day thru Week 52) and the other (Patient 2) initiated allopurinol 2 weeks after discontinuing pegloticase+methotrexate (150 mg/day until Week 47, 300 mg/day thru Week 52).
