# Supplementary material for: A multicentre, efficacy and safety study of methotrexate to increase response rates in patients with uncontrolled gout receiving pegloticase (MIRROR): 12-month efficacy, safety, immunogenicity, and pharmacokinetic findings during long-term extension of an open-label study
Source: Arthritis Res Ther. 2022 Aug 25;24:208. doi: 10.1186/s13075-022-02865-z (PMC9404640; doi:10.1186/s13075-022-02865-z)
Supplement: Supplementary file 3 — Additional file 3: Supplemental Figure 2. Individual concentration-time course of pegloticase and MTX polyglutamates. [file 13075_2022_2865_MOESM3_ESM.pdf]

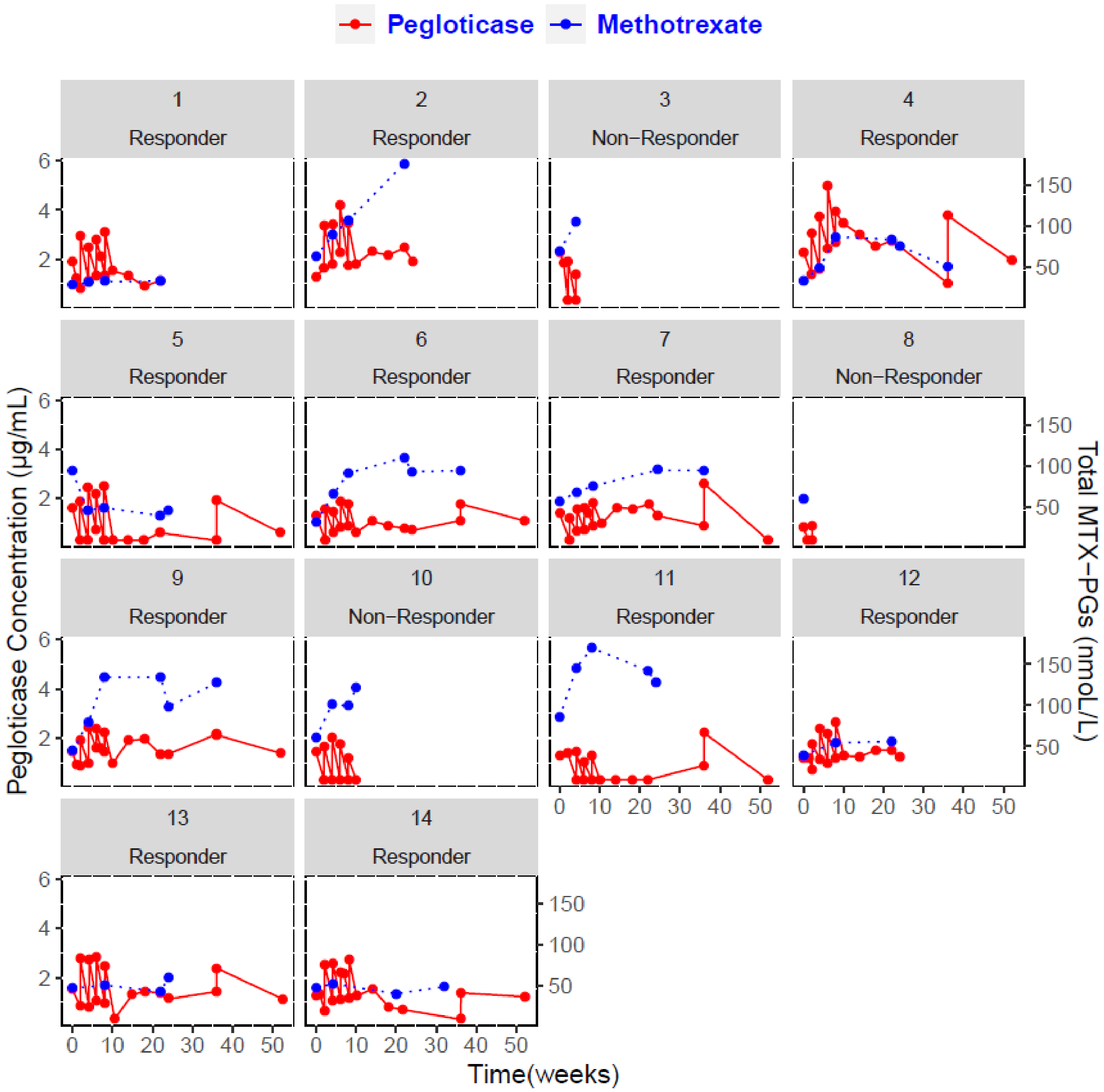

Supplemental Figure 2. Individual concentration-time course of pegloticase and MTX polyglutamates.
